# 1-(4-Chloro­phen­yl)-2-(1,3-diazepan-2-yl­idene)ethanone

**DOI:** 10.1107/S1600536813013858

**Published:** 2013-05-25

**Authors:** Xiao-Wei Zhang, Jian-Hui Xia, Zhao-Hui Xu, Li-Ben Wang, Chu-Yi Yu

**Affiliations:** a College of Chemistry and Chemical Engineering, Jiang Xi Normal University, Nanchang, Jiang Xi 330022, People’s Republic of China; b Beijing National Laboratory for Molecular Science (BNLMS), CAS Key Laboratory of Molecular Recognition and Function, Institute of Chemistry, Chinese Academy of Sciences, Beijing 100190, People’s Republic of China

## Abstract

In the title compound, C_13_H_15_ClN_2_O, there are two crystallographically independent but conformationally similar (*E*)-mol­ecules in the asymmetric unit [dihedral angles between the phenyl ring and a common planar fragment of the 1,3-diazepane moiety = 47.34 (16) and 48.00 (16)°]. The seven-membered ring system adopts a chair conformation in both molecules. In the crystal, N—H⋯O hydrogen bonds lead to chains extending along the *b*-axis direction.

## Related literature

Heterocyclic ketene aminals are a useful synthon for the synthesis of fused heterocycles, see: Huang & Wang (1994 ▶). For their bioactivity and potential applications as pesticides and in medicine, see: Kondo *et al.* (1990 ▶); Jordan *et al.* (2002 ▶). For the synthesis, see: Huang & Liu (1989 ▶). For a similar structure, see: Yu *et al.* (2006 ▶).
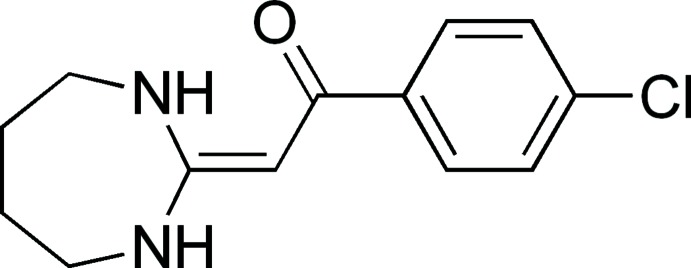


## Experimental

### 

#### Crystal data

C_13_H_15_ClN_2_O
*M* *_r_* = 250.72
Triclinic, 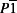

*a* = 10.779 (4) Å
*b* = 10.815 (3) Å
*c* = 12.158 (4) Å
α = 96.091 (4)°
β = 110.710 (4)°
γ = 101.244 (4)°
*V* = 1276.4 (7) Å^3^
*Z* = 4
Mo *K*α radiation
μ = 0.29 mm^−1^
*T* = 173 K
0.25 × 0.15 × 0.14 mm

#### Data collection

Rigaku Saturn724+ CCD diffractometer
Absorption correction: multi-scan (*CrystalClear*; Rigaku, 2007 ▶) *T*_min_ = 0.606, *T*_max_ = 1.000
11344 measured reflections
5780 independent reflections
4854 reflections with *I* > 2σ(*I*)
*R*_int_ = 0.048

#### Refinement

*R*[*F* ^2^ > 2σ(*F* ^2^)] = 0.075
*wR*(*F* ^2^) = 0.162
*S* = 1.12
5780 reflections
307 parameters
H-atom parameters constrained
Δρ_max_ = 0.49 e Å^−3^
Δρ_min_ = −0.42 e Å^−3^

### 

Data collection: *CrystalClear* (Rigaku, 2007 ▶); cell refinement: *CrystalClear*; data reduction: *CrystalClear*; program(s) used to solve structure: *SHELXS97* (Sheldrick, 2008 ▶); program(s) used to refine structure: *SHELXL97* (Sheldrick, 2008 ▶); molecular graphics: *OLEX2* (Dolomanov *et al.*, 2009 ▶); software used to prepare material for publication: *SHELXL97*.

## Supplementary Material

Crystal structure: contains datablock(s) I, global. DOI: 10.1107/S1600536813013858/zs2253sup1.cif

Structure factors: contains datablock(s) I. DOI: 10.1107/S1600536813013858/zs2253Isup2.hkl

Supplementary material file. DOI: 10.1107/S1600536813013858/zs2253Isup3.cml

Additional supplementary materials:  crystallographic information; 3D view; checkCIF report

## Figures and Tables

**Table 1:** Hydrogen-bond geometry (Å, °)

*D*—H⋯*A*	*D*—H	H⋯*A*	*D*⋯*A*	*D*—H⋯*A*
N2—H2⋯O2	0.88	2.07	2.834 (3)	145
N3—H3⋯O1^i^	0.88	2.09	2.878 (3)	149

Symmetry code: (i) 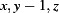.

**Fig. 1.**
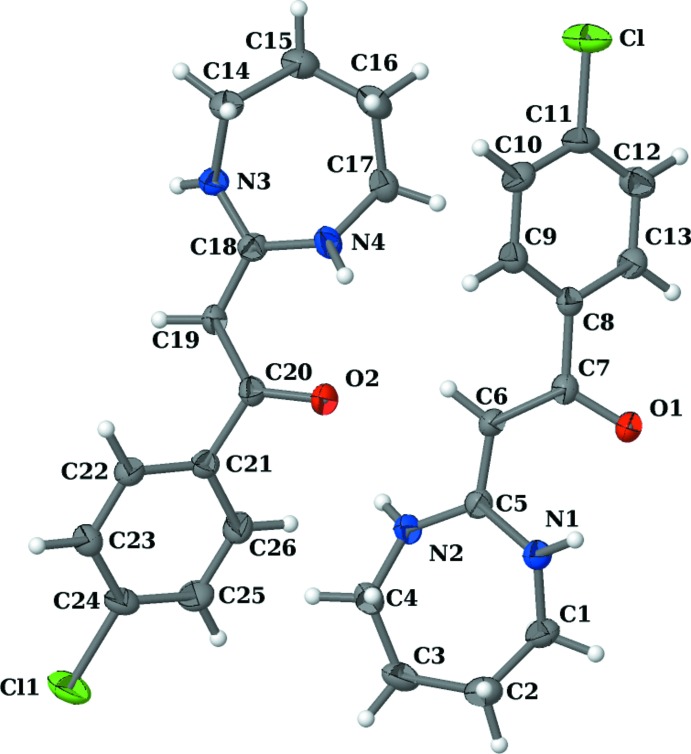
The molecular structure and atom numbering scheme for the two independent molecules in the asymmetric unit of the title compound. Displacement ellipsoids are drawn at the 30% probability level and H atoms are shown as spheres of arbitrary radius.

**Fig. 2.**
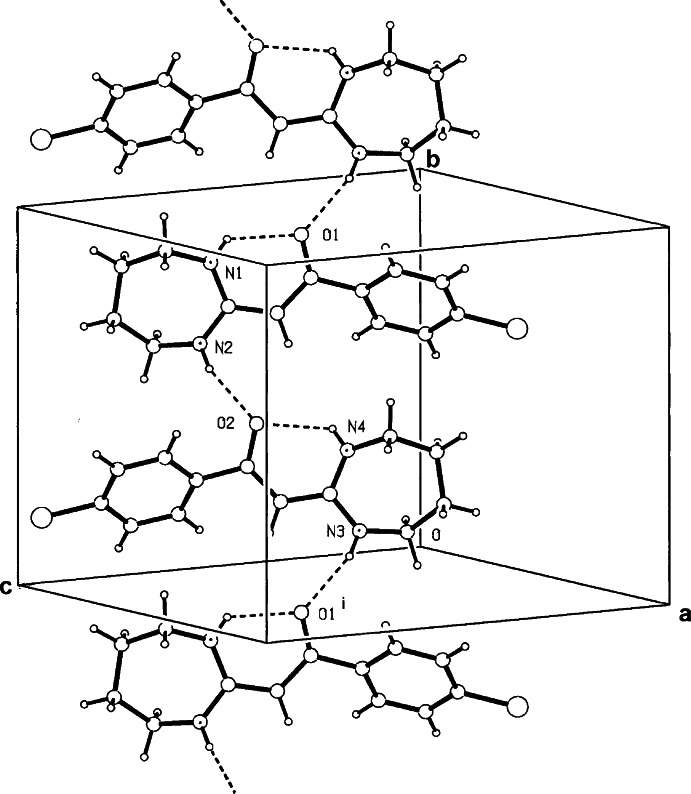
The packing of the title compound in the unit cell. Dashed lines indicate N—H···O hydrogen bonds and intramolecular N—H···O interactions. For symmetry code (i), see Table 1.

## supplementary crystallographic information

### Comment

Heterocyclic ketene aminals are viewed as a useful synthon for the synthesis of fused heterocycles (Huang & Wang, 1994). Also, some of these compounds display certain bioactivities and show potential for applications in the fields of pesticides and medicine, e.g. as antimicrobial reagents (Kondo *et al.*, 1990) and anti-anxiety reagents (Jordan *et al.*, 2002). In the title compound, C_13_H_15_ClN_2_O, there are two crystallographically independent but similar (*E*)-molecules in the asymmetric unit (Fig. 1), with the dihedral angle between the phenyl ring and the ethylenic moiety [(C8–C13) and N1, N2, C5, C6 in molecule 1 and (C21–C26) and (N3, N4, C18, C19) in molecule 2] being 47.34 (16) and 48.00 (16)°, respectively. Intramolecular interactions (N1—H···O1 in molecule 1 and N4—H···O2 in molecule 2) (Table 1) stabilize the conformations. The seven-membered ring adopts a stable chair conformation. In the crystal, intermolecular N—H···O hydrogen-bonding interactions (Table 1) give one-dimensional chains which extend down *b* (Fig. 2).

### Experimental

The title compound was prepared according to the method of Huang & Liu (1989). m.p. 448–450 K. MS: m/*z* = 250 (*M*^+^). IR: 3180 (NH), 1630 (C=O), 1578 cm^-l^ (C=C). ^1^H-NMR: *δ* = 7.70 (d, 2H), 7.40 (d, 2H), 11.13 (s, lH), 7.10 (s, lH), 5.27 (s, lH), 3.05–3.30 (m, 4H), 1.55–1.72 (m, 4H) p.p.m.. ^13^C-NMR: *δ* = 179.7, 168.4, 140.2, 133.4, 127.7, 127.4, 79.1, 43.8, 27.6 p.p.m.. Anal. calc. for C_13_H_15_ClN_2_O: C, 62.27; H, 6.03; N, 11.18. Found C, 62.09; H, 5.94; N, 11.02.

### Refinement

H atoms were positioned geometrically and refined using a riding model, with C—H = 0.99 Å (methylene) or 0.95 Å (aromatic) and N—H = 0.88 Å (N), with *U*_iso_(H) = 1.5*U*_eq_(C) (methylene) or *U*_iso_(H) = 1.2 *U*_eq_(aromatic C or N).

### Crystal data

**Table:**

C_13_H_15_ClN_2_O	*Z* = 4
*M**_r_* = 250.72	*F*(000) = 528
Triclinic, *P*1	*D*_x_ = 1.305 Mg m^−^^3^
Hall symbol: -P 1	Melting point = 448–450 K
*a* = 10.779 (4) Å	Mo *K*α radiation, λ = 0.71073 Å
*b* = 10.815 (3) Å	Cell parameters from 3768 reflections
*c* = 12.158 (4) Å	θ = 1.8–27.5°
α = 96.091 (4)°	µ = 0.29 mm^−^^1^
β = 110.710 (4)°	*T* = 173 K
γ = 101.244 (4)°	Block, white
*V* = 1276.4 (7) Å^3^	0.25 × 0.15 × 0.14 mm

### Data collection

**Table:**

Rigaku Saturn724+ CCD diffractometer	5780 independent reflections
Radiation source: sealed tube	4854 reflections with *I* > 2σ(*I*)
Graphite monochromator	*R*_int_ = 0.048
ω scans at fixed χ = 45°	θ_max_ = 27.5°, θ_min_ = 2.1°
Absorption correction: multi-scan (*CrystalClear*; Rigaku, 2007)	*h* = −13→13
*T*_min_ = 0.606, *T*_max_ = 1.000	*k* = −14→13
11344 measured reflections	*l* = −15→15

### Refinement

**Table:**

Refinement on *F*^2^	Primary atom site location: structure-invariant direct methods
Least-squares matrix: full	Secondary atom site location: difference Fourier map
*R*[*F*^2^ > 2σ(*F*^2^)] = 0.075	Hydrogen site location: inferred from neighbouring sites
*wR*(*F*^2^) = 0.162	H-atom parameters constrained
*S* = 1.12	*w* = 1/[σ^2^(*F*_o_^2^) + (0.0466*P*)^2^ + 1.3126*P*] where *P* = (*F*_o_^2^ + 2*F*_c_^2^)/3
5780 reflections	(Δ/σ)_max_ < 0.001
307 parameters	Δρ_max_ = 0.49 e Å^−^^3^
0 restraints	Δρ_min_ = −0.42 e Å^−^^3^

### Special details

**Table:**

Geometry. All e.s.d.'s (except the e.s.d. in the dihedral angle between two l.s. planes) are estimated using the full covariance matrix. The cell e.s.d.'s are taken into account individually in the estimation of e.s.d.'s in distances, angles and torsion angles; correlations between e.s.d.'s in cell parameters are only used when they are defined by crystal symmetry. An approximate (isotropic) treatment of cell e.s.d.'s is used for estimating e.s.d.'s involving l.s. planes.
Refinement. Refinement of *F*^2^ against ALL reflections. The weighted *R*-factor *wR* and goodness of fit *S* are based on *F*^2^, conventional *R*-factors *R* are based on *F*, with *F* set to zero for negative *F*^2^. The threshold expression of *F*^2^ > σ(*F*^2^) is used only for calculating *R*-factors(gt) *etc*. and is not relevant to the choice of reflections for refinement. *R*-factors based on *F*^2^ are statistically about twice as large as those based on *F*, and *R*- factors based on ALL data will be even larger.

### Fractional atomic coordinates and isotropic or equivalent isotropic displacement parameters (Å^2^)

**Table:**

	*x*	*y*	*z*	*U*_iso_*/*U*_eq_	
Cl1	0.01481 (9)	0.17456 (9)	0.95043 (7)	0.0515 (2)	
Cl	0.43184 (9)	0.64786 (9)	0.02814 (7)	0.0516 (2)	
O1	0.3033 (2)	0.92241 (17)	0.48282 (17)	0.0311 (4)	
O2	0.2480 (2)	0.42103 (17)	0.56002 (18)	0.0335 (5)	
N1	0.1989 (2)	0.8486 (2)	0.6444 (2)	0.0305 (5)	
H1	0.1868	0.9035	0.5954	0.037*	
N2	0.1648 (2)	0.6291 (2)	0.6502 (2)	0.0315 (5)	
H2	0.2118	0.5704	0.6555	0.038*	
N3	0.3217 (2)	0.1261 (2)	0.3506 (2)	0.0305 (5)	
H3	0.3486	0.0677	0.3921	0.037*	
N4	0.3187 (2)	0.3421 (2)	0.3784 (2)	0.0304 (5)	
H4	0.2633	0.3913	0.3810	0.036*	
C1	0.2192 (3)	0.8941 (3)	0.7685 (3)	0.0360 (7)	
H1A	0.2364	0.9889	0.7827	0.043*	
H1B	0.3023	0.8726	0.8219	0.043*	
C2	0.1006 (3)	0.8396 (3)	0.8037 (3)	0.0414 (7)	
H2A	0.0144	0.8483	0.7429	0.050*	
H2B	0.1138	0.8906	0.8813	0.050*	
C3	0.0865 (4)	0.6990 (3)	0.8145 (3)	0.0413 (7)	
H3A	0.1724	0.6903	0.8755	0.050*	
H3B	0.0117	0.6713	0.8427	0.050*	
C4	0.0566 (3)	0.6115 (3)	0.6977 (3)	0.0343 (6)	
H4A	0.0362	0.5216	0.7089	0.041*	
H4B	−0.0273	0.6239	0.6366	0.041*	
C5	0.1976 (3)	0.7281 (2)	0.5993 (2)	0.0275 (6)	
C6	0.2322 (3)	0.7060 (2)	0.4985 (2)	0.0268 (5)	
H6	0.2198	0.6194	0.4635	0.032*	
C7	0.2834 (3)	0.8023 (2)	0.4467 (2)	0.0258 (5)	
C8	0.3180 (3)	0.7624 (2)	0.3414 (2)	0.0259 (5)	
C9	0.2356 (3)	0.6569 (3)	0.2512 (3)	0.0327 (6)	
H9	0.1552	0.6079	0.2564	0.039*	
C10	0.2687 (3)	0.6221 (3)	0.1543 (3)	0.0359 (6)	
H10	0.2111	0.5508	0.0927	0.043*	
C11	0.3869 (3)	0.6927 (3)	0.1485 (2)	0.0337 (6)	
C12	0.4705 (3)	0.7977 (3)	0.2366 (3)	0.0365 (7)	
H12	0.5520	0.8451	0.2319	0.044*	
C13	0.4345 (3)	0.8334 (3)	0.3316 (2)	0.0314 (6)	
H13	0.4901	0.9073	0.3908	0.038*	
C14	0.3117 (3)	0.1092 (3)	0.2266 (3)	0.0354 (6)	
H14A	0.2231	0.1235	0.1755	0.042*	
H14B	0.3110	0.0192	0.2005	0.042*	
C15	0.4259 (4)	0.1975 (3)	0.2056 (3)	0.0441 (8)	
H15A	0.4211	0.1679	0.1239	0.053*	
H15B	0.5153	0.1917	0.2634	0.053*	
C16	0.4181 (4)	0.3370 (3)	0.2190 (3)	0.0467 (8)	
H16A	0.4910	0.3887	0.1993	0.056*	
H16B	0.3287	0.3428	0.1612	0.056*	
C17	0.4338 (3)	0.3927 (3)	0.3444 (3)	0.0353 (6)	
H17A	0.5178	0.3768	0.4022	0.042*	
H17B	0.4473	0.4869	0.3519	0.042*	
C18	0.2926 (3)	0.2248 (2)	0.4058 (2)	0.0267 (5)	
C19	0.2356 (3)	0.2047 (2)	0.4922 (2)	0.0274 (5)	
H19	0.2061	0.1188	0.5006	0.033*	
C20	0.2198 (3)	0.3026 (2)	0.5660 (2)	0.0266 (5)	
C21	0.1675 (3)	0.2680 (2)	0.6605 (2)	0.0258 (5)	
C22	0.0618 (3)	0.1599 (3)	0.6392 (2)	0.0299 (6)	
H22	0.0222	0.1047	0.5628	0.036*	
C23	0.0134 (3)	0.1315 (3)	0.7275 (3)	0.0321 (6)	
H23	−0.0601	0.0587	0.7118	0.039*	
C24	0.0741 (3)	0.2111 (3)	0.8384 (2)	0.0332 (6)	
C25	0.1789 (3)	0.3185 (3)	0.8625 (3)	0.0392 (7)	
H25	0.2196	0.3719	0.9398	0.047*	
C26	0.2243 (3)	0.3477 (3)	0.7732 (3)	0.0351 (6)	
H26	0.2950	0.4229	0.7887	0.042*	

### Atomic displacement parameters (Å^2^)

**Table:**

	*U*^11^	*U*^22^	*U*^33^	*U*^12^	*U*^13^	*U*^23^
Cl1	0.0604 (5)	0.0674 (6)	0.0365 (4)	0.0123 (4)	0.0303 (4)	0.0159 (4)
Cl	0.0496 (5)	0.0742 (6)	0.0351 (4)	0.0167 (4)	0.0236 (4)	−0.0008 (4)
O1	0.0418 (11)	0.0198 (9)	0.0341 (10)	0.0049 (8)	0.0194 (9)	0.0035 (8)
O2	0.0467 (12)	0.0222 (9)	0.0404 (11)	0.0098 (8)	0.0253 (10)	0.0091 (8)
N1	0.0413 (13)	0.0250 (11)	0.0303 (12)	0.0103 (10)	0.0179 (10)	0.0077 (10)
N2	0.0431 (14)	0.0246 (11)	0.0374 (13)	0.0120 (10)	0.0243 (11)	0.0116 (10)
N3	0.0437 (14)	0.0269 (11)	0.0280 (12)	0.0122 (10)	0.0195 (10)	0.0078 (9)
N4	0.0349 (12)	0.0264 (11)	0.0383 (13)	0.0115 (10)	0.0196 (11)	0.0144 (10)
C1	0.0420 (17)	0.0338 (15)	0.0312 (15)	0.0068 (13)	0.0169 (13)	−0.0027 (12)
C2	0.0494 (19)	0.0418 (17)	0.0387 (17)	0.0122 (14)	0.0251 (15)	0.0019 (14)
C3	0.0521 (19)	0.0480 (18)	0.0365 (16)	0.0151 (15)	0.0293 (15)	0.0124 (14)
C4	0.0371 (15)	0.0328 (15)	0.0395 (16)	0.0073 (12)	0.0215 (13)	0.0120 (13)
C5	0.0281 (13)	0.0233 (12)	0.0318 (14)	0.0061 (10)	0.0129 (11)	0.0036 (11)
C6	0.0343 (14)	0.0194 (12)	0.0286 (13)	0.0064 (10)	0.0149 (11)	0.0022 (10)
C7	0.0257 (12)	0.0226 (12)	0.0274 (13)	0.0047 (10)	0.0090 (10)	0.0049 (10)
C8	0.0308 (14)	0.0217 (12)	0.0269 (13)	0.0063 (10)	0.0128 (11)	0.0062 (10)
C9	0.0355 (15)	0.0280 (14)	0.0337 (15)	0.0016 (11)	0.0163 (12)	0.0035 (12)
C10	0.0408 (16)	0.0325 (15)	0.0298 (14)	0.0049 (12)	0.0130 (12)	−0.0026 (12)
C11	0.0411 (16)	0.0417 (16)	0.0250 (13)	0.0176 (13)	0.0168 (12)	0.0061 (12)
C12	0.0305 (14)	0.0448 (17)	0.0355 (15)	0.0061 (12)	0.0165 (12)	0.0056 (13)
C13	0.0312 (14)	0.0295 (14)	0.0298 (14)	0.0032 (11)	0.0108 (11)	0.0015 (11)
C14	0.0441 (17)	0.0347 (15)	0.0288 (14)	0.0089 (13)	0.0170 (13)	0.0034 (12)
C15	0.055 (2)	0.0457 (18)	0.0421 (18)	0.0109 (15)	0.0318 (16)	0.0065 (15)
C16	0.062 (2)	0.0445 (18)	0.0451 (19)	0.0070 (16)	0.0354 (17)	0.0141 (15)
C17	0.0343 (15)	0.0319 (15)	0.0404 (16)	0.0018 (12)	0.0188 (13)	0.0068 (13)
C18	0.0283 (13)	0.0266 (13)	0.0251 (13)	0.0081 (10)	0.0094 (11)	0.0047 (11)
C19	0.0345 (14)	0.0201 (12)	0.0318 (14)	0.0067 (10)	0.0170 (12)	0.0078 (11)
C20	0.0315 (13)	0.0221 (12)	0.0271 (13)	0.0064 (10)	0.0120 (11)	0.0068 (10)
C21	0.0286 (13)	0.0246 (12)	0.0265 (13)	0.0091 (10)	0.0117 (11)	0.0058 (10)
C22	0.0353 (14)	0.0265 (13)	0.0279 (13)	0.0059 (11)	0.0139 (11)	0.0024 (11)
C23	0.0337 (15)	0.0301 (14)	0.0341 (15)	0.0049 (11)	0.0162 (12)	0.0075 (12)
C24	0.0381 (16)	0.0408 (16)	0.0273 (14)	0.0123 (13)	0.0182 (12)	0.0092 (12)
C25	0.0468 (18)	0.0394 (16)	0.0261 (14)	0.0049 (14)	0.0128 (13)	−0.0013 (12)
C26	0.0405 (16)	0.0288 (14)	0.0302 (15)	−0.0005 (12)	0.0131 (13)	0.0006 (12)

### Geometric parameters (Å, º)

**Table:**

Cl1—C24	1.747 (3)	C9—C10	1.383 (4)
Cl—C11	1.747 (3)	C9—H9	0.9500
O1—C7	1.277 (3)	C10—C11	1.381 (4)
O2—C20	1.273 (3)	C10—H10	0.9500
N1—C5	1.354 (3)	C11—C12	1.382 (4)
N1—C1	1.461 (3)	C12—C13	1.383 (4)
N1—H1	0.8800	C12—H12	0.9500
N2—C5	1.344 (3)	C13—H13	0.9500
N2—C4	1.463 (3)	C14—C15	1.519 (4)
N2—H2	0.8800	C14—H14A	0.9900
N3—C18	1.347 (3)	C14—H14B	0.9900
N3—C14	1.461 (3)	C15—C16	1.522 (5)
N3—H3	0.8800	C15—H15A	0.9900
N4—C18	1.348 (3)	C15—H15B	0.9900
N4—C17	1.468 (3)	C16—C17	1.515 (4)
N4—H4	0.8800	C16—H16A	0.9900
C1—C2	1.520 (4)	C16—H16B	0.9900
C1—H1A	0.9900	C17—H17A	0.9900
C1—H1B	0.9900	C17—H17B	0.9900
C2—C3	1.522 (4)	C18—C19	1.407 (4)
C2—H2A	0.9900	C19—C20	1.390 (4)
C2—H2B	0.9900	C19—H19	0.9500
C3—C4	1.513 (4)	C20—C21	1.499 (4)
C3—H3A	0.9900	C21—C22	1.392 (4)
C3—H3B	0.9900	C21—C26	1.395 (4)
C4—H4A	0.9900	C22—C23	1.387 (4)
C4—H4B	0.9900	C22—H22	0.9500
C5—C6	1.412 (4)	C23—C24	1.377 (4)
C6—C7	1.396 (4)	C23—H23	0.9500
C6—H6	0.9500	C24—C25	1.376 (4)
C7—C8	1.499 (4)	C25—C26	1.381 (4)
C8—C13	1.390 (4)	C25—H25	0.9500
C8—C9	1.394 (4)	C26—H26	0.9500
			
C5—N1—C1	124.8 (2)	C11—C12—C13	119.4 (3)
C5—N1—H1	117.6	C11—C12—H12	120.3
C1—N1—H1	117.6	C13—C12—H12	120.3
C5—N2—C4	124.8 (2)	C12—C13—C8	120.8 (3)
C5—N2—H2	117.6	C12—C13—H13	119.6
C4—N2—H2	117.6	C8—C13—H13	119.6
C18—N3—C14	124.7 (2)	N3—C14—C15	115.0 (2)
C18—N3—H3	117.7	N3—C14—H14A	108.5
C14—N3—H3	117.7	C15—C14—H14A	108.5
C18—N4—C17	124.0 (2)	N3—C14—H14B	108.5
C18—N4—H4	118.0	C15—C14—H14B	108.5
C17—N4—H4	118.0	H14A—C14—H14B	107.5
N1—C1—C2	115.2 (2)	C14—C15—C16	112.7 (3)
N1—C1—H1A	108.5	C14—C15—H15A	109.1
C2—C1—H1A	108.5	C16—C15—H15A	109.1
N1—C1—H1B	108.5	C14—C15—H15B	109.1
C2—C1—H1B	108.5	C16—C15—H15B	109.1
H1A—C1—H1B	107.5	H15A—C15—H15B	107.8
C1—C2—C3	113.2 (3)	C17—C16—C15	112.6 (3)
C1—C2—H2A	108.9	C17—C16—H16A	109.1
C3—C2—H2A	108.9	C15—C16—H16A	109.1
C1—C2—H2B	108.9	C17—C16—H16B	109.1
C3—C2—H2B	108.9	C15—C16—H16B	109.1
H2A—C2—H2B	107.8	H16A—C16—H16B	107.8
C4—C3—C2	113.0 (2)	N4—C17—C16	115.5 (3)
C4—C3—H3A	109.0	N4—C17—H17A	108.4
C2—C3—H3A	109.0	C16—C17—H17A	108.4
C4—C3—H3B	109.0	N4—C17—H17B	108.4
C2—C3—H3B	109.0	C16—C17—H17B	108.4
H3A—C3—H3B	107.8	H17A—C17—H17B	107.5
N2—C4—C3	116.3 (2)	N3—C18—N4	119.9 (2)
N2—C4—H4A	108.2	N3—C18—C19	119.7 (2)
C3—C4—H4A	108.2	N4—C18—C19	120.4 (2)
N2—C4—H4B	108.2	C20—C19—C18	124.1 (2)
C3—C4—H4B	108.2	C20—C19—H19	117.9
H4A—C4—H4B	107.4	C18—C19—H19	117.9
N2—C5—N1	120.6 (2)	O2—C20—C19	124.6 (2)
N2—C5—C6	119.4 (2)	O2—C20—C21	117.1 (2)
N1—C5—C6	120.0 (2)	C19—C20—C21	118.4 (2)
C7—C6—C5	124.6 (2)	C22—C21—C26	118.6 (2)
C7—C6—H6	117.7	C22—C21—C20	122.2 (2)
C5—C6—H6	117.7	C26—C21—C20	119.2 (2)
O1—C7—C6	124.6 (2)	C23—C22—C21	121.1 (3)
O1—C7—C8	117.5 (2)	C23—C22—H22	119.5
C6—C7—C8	117.9 (2)	C21—C22—H22	119.5
C13—C8—C9	118.4 (2)	C24—C23—C22	118.6 (3)
C13—C8—C7	119.4 (2)	C24—C23—H23	120.7
C9—C8—C7	122.1 (2)	C22—C23—H23	120.7
C10—C9—C8	121.3 (3)	C25—C24—C23	121.8 (3)
C10—C9—H9	119.4	C25—C24—Cl1	119.5 (2)
C8—C9—H9	119.4	C23—C24—Cl1	118.7 (2)
C11—C10—C9	118.9 (3)	C24—C25—C26	119.2 (3)
C11—C10—H10	120.6	C24—C25—H25	120.4
C9—C10—H10	120.6	C26—C25—H25	120.4
C10—C11—C12	121.1 (3)	C25—C26—C21	120.7 (3)
C10—C11—Cl	119.4 (2)	C25—C26—H26	119.6
C12—C11—Cl	119.5 (2)	C21—C26—H26	119.6
			
C5—N1—C1—C2	−70.7 (4)	C18—N3—C14—C15	75.0 (4)
N1—C1—C2—C3	72.1 (4)	N3—C14—C15—C16	−69.8 (4)
C1—C2—C3—C4	−62.4 (4)	C14—C15—C16—C17	62.4 (4)
C5—N2—C4—C3	−73.6 (4)	C18—N4—C17—C16	74.6 (4)
C2—C3—C4—N2	65.7 (4)	C15—C16—C17—N4	−69.9 (4)
C4—N2—C5—N1	39.6 (4)	C14—N3—C18—N4	−33.7 (4)
C4—N2—C5—C6	−141.0 (3)	C14—N3—C18—C19	146.5 (3)
C1—N1—C5—N2	24.8 (4)	C17—N4—C18—N3	−33.2 (4)
C1—N1—C5—C6	−154.5 (3)	C17—N4—C18—C19	146.6 (3)
N2—C5—C6—C7	−172.1 (3)	N3—C18—C19—C20	170.6 (3)
N1—C5—C6—C7	7.3 (4)	N4—C18—C19—C20	−9.2 (4)
C5—C6—C7—O1	−1.1 (4)	C18—C19—C20—O2	3.9 (4)
C5—C6—C7—C8	178.7 (2)	C18—C19—C20—C21	−175.3 (2)
O1—C7—C8—C13	39.3 (4)	O2—C20—C21—C22	139.5 (3)
C6—C7—C8—C13	−140.5 (3)	C19—C20—C21—C22	−41.2 (4)
O1—C7—C8—C9	−139.7 (3)	O2—C20—C21—C26	−39.2 (4)
C6—C7—C8—C9	40.5 (4)	C19—C20—C21—C26	140.1 (3)
C13—C8—C9—C10	0.5 (4)	C26—C21—C22—C23	0.1 (4)
C7—C8—C9—C10	179.5 (3)	C20—C21—C22—C23	−178.6 (3)
C8—C9—C10—C11	1.0 (4)	C21—C22—C23—C24	−1.4 (4)
C9—C10—C11—C12	−1.0 (5)	C22—C23—C24—C25	1.3 (4)
C9—C10—C11—Cl	178.7 (2)	C22—C23—C24—Cl1	−179.2 (2)
C10—C11—C12—C13	−0.6 (5)	C23—C24—C25—C26	0.3 (5)
Cl—C11—C12—C13	179.8 (2)	Cl1—C24—C25—C26	−179.3 (2)
C11—C12—C13—C8	2.1 (4)	C24—C25—C26—C21	−1.6 (5)
C9—C8—C13—C12	−2.1 (4)	C22—C21—C26—C25	1.5 (4)
C7—C8—C13—C12	178.9 (3)	C20—C21—C26—C25	−179.8 (3)

### Hydrogen-bond geometry (Å, º)

**Table:**

*D*—H···*A*	*D*—H	H···*A*	*D*···*A*	*D*—H···*A*
N1—H1···O1	0.88	2.16	2.704 (3)	119
N2—H2···O2	0.88	2.07	2.834 (3)	145
N3—H3···O1^i^	0.88	2.09	2.878 (3)	149
N4—H4···O2	0.88	2.23	2.689 (3)	112
C6—H6···O2	0.95	2.57	3.270 (3)	131
C13—H13···O1^ii^	0.95	2.47	3.372 (4)	159
C19—H19···O1^i^	0.95	2.57	3.274 (3)	132

Symmetry codes: (i) *x*, *y*−1, *z*; (ii) −*x*+1, −*y*+2, −*z*+1.
